# Social Nesting, Animal Welfare, and Disease Monitoring

**DOI:** 10.3390/ani11041079

**Published:** 2021-04-09

**Authors:** Lydia Giménez-Llort, Virginia Torres-Lista

**Affiliations:** 1 Institut de Neurociències, Universitat Autònoma de Barcelona, E-08193 Barcelona, Spain; virginiatorreslista@yahoo.es; 2 Department of Psychiatry and Forensic Medicine, School of Medicine, Universitat Autònoma de Barcelona, E-08193 Barcelona, Spain

**Keywords:** nest-building, social behavior, behavioral monitoring, animal welfare, 3xTg-AD mice, Alzheimer’s disease, gender medicine, early-life events, early-life interventions, long-term effects

## Abstract

**Simple Summary:**

Most standardized tools to evaluate welfare and disease progression in animals assess the individuals, while social behaviors are scarcely monitored, despite being useful to detecting acute illness and chronic and mental health problems. The main reason is that social behavior is complex and time-consuming. We are currently using the nests built by animals living together, a species-typical behavior naturally occurring in standard housing conditions, to monitor them. Here, we provide an example of its use to evaluate social deficits and the long-term effects of a neonatal tactile-proprioceptive sensorial treatment from postnatal day 1 to 21, in male and female adult mice modeling Alzheimer’s disease compared to mice with normal aging. Social nesting was worse in the mutants, mostly in males, since the number of days needed to build a perfect nest was longer or unsuccessful in a three-day test. Early life intervention was successful. Social nesting, easily included in housing routines, can be a useful tool to assess animal welfare, monitor disease progress, and evaluate potential risk factors and effects of preventive/therapeutical strategies. Other advantages, such as being a noninvasive, painless, simple, short, and low-cost, rend social nesting feasible to be implemented in most animal department settings.

**Abstract:**

The assessment of welfare and disease progression in animal models is critical. Most tools rely on evaluating individual subjects, whereas social behaviors, also sensitive to acute illness, chronic diseases, or mental health, are scarcely monitored because they are complex and time-consuming. We propose the evaluation of social nesting, a species-typical behavior naturally occurring in standard housing conditions, for such behavioral monitoring. We provide an example of its use to evaluate social deficits and the long-term effects of neonatal tactile-proprioceptive sensorial stimulation from postnatal day 1 to 21, in male and female adult 3xTg-AD mice for Alzheimer’s disease compared to sex- and age-matched non-transgenic (NTg) counterparts with normal aging. Social nesting was sensitive to genotype (worse in 3xTg-AD mice), sex (worse in males), profile, and treatment (distinct time to observe the maximum score and incidence of the perfect nest). Since social nesting can be easily included in housing routines, this neuroethological approach can be useful for animal welfare, monitoring the disease’s progress, and evaluating potential risk factors and effects of preventive/therapeutical strategies. Finally, the noninvasive, painless, simple, short time, and low-cost features of this home-cage monitoring are advantages that make social nesting feasible to be successfully implemented in most animal department settings.

## 1. Introduction

Assessing animals’ wellbeing, disease progression, and effects of treatments would benefit from home-cage noninterventional tools for behavioral phenotyping and monitoring. The home-cage also allows translational studies on social deficits known to impact patients and caregivers in Alzheimer’s disease (AD) or mental health disorders such as schizophrenia, depression, and autism [1,2]. On the other hand, modulatory effects of social factors on physical and mental health are well-known, whereas certain disruptive social conditions are considered triggers or precipitators of dementia symptoms [3,4]. In this sense, species-typical behaviors naturally occurring in standard housing conditions such as nest-building [5] could help identify acute illness, monitor disease progression, and assess animal welfare [6,7,8,9].

Although nest-building primarily aims to provide protection, facilitation of family structure, and maternal interaction, this ethological behavior is also exhibited by male and female adults meant for thermoregulation, being sensitive to environmental conditions [10]. Nest-building is also considered an indicator of animal wellbeing [11] and useful for identifying ill mice [9]. Conversely, animal welfare guidelines indicate providing animals with nesting material a must to improve their housing conditions [12]. We were first to report impairment in nesting behavior in old animals and its worsening in 3xTg-AD mice at ages mimicking early (6 months) and advanced (12 months) stages of Alzheimer’s disease [7]. We described deficiencies in this instrumental task considered mimicking the “assessment of motor and processes skills” used for daily life activities in older people and the progressive functional impairment observed in the AD patient [13]. A three-day assessment worked better than standard protocols assessing only at 24 h to unveil genotype-, sex- and age-dependent differences. Additionally, 3xTg-AD mice showed a substantial delay in approaching the nesting material, with increased fear, apathy, or attentional deficits as putative underlying behavioral constructs. However, single housing can be less than optimal from an ethical and ethological perspective and not appropriate in many experimental studies, including those for drug screening, long-term monitoring, or assessment of nonpharmacological interventions.

The present brief report aims to provide proof-of-concept for social-nesting as an animal’s welfare neuroethological tool that can monitor functional impairment in aging and neurogenerative disease processes and assess disease modulation by chronic or long-term treatments. In this respect, we already demonstrated that social nesting impairment, studied as a species-specific affiliative social behavior, can be detected in breeding schedules [7]. In our recent scientific report, old female 3xTg-AD mice showed less nest-building social collaboration skills than wild-type groups, whereas Se treatment increased their nesting activity and reversed other behavioral impairments and neuropathology [14]. Here, we analyzed social nesting in 80 animals, 24 social groups of six-month-old male and female 3xTg-AD mice exhibiting enhanced bizarre or disruptive behaviors compared to counterparts [15]. In agreement with profound and long-lasting neurobiological, cognitive, and behavioral effects of neonatal handling in rodents [16,17,18], the long-term success of this intervention administered during the ontogeny was demonstrated on those 80 animals [15]. While nothing was known on neonatal handling’s social effects, here we show that the dysfunctional patterns of male and female 3xTg-AD mice in social nesting under standard home-cage conditions could be used for early social endophenotype monitoring and to study the effects of that long-term treatment.

## 2. Materials and Methods

### 2.1. Subjects

A total of 80 animals (24 cages, 3–4 per cage), 40 males and 40 females from Spanish colonies [19] of homozygous non-transgenic (NTg) and 3xTg-AD mice genetically engineered at the University of California Irvine, in a hybrid C57BL/6J × 129/Sv genetic background [20,21] were used. Animals were maintained in Macrolon cages (open cages, 35 × 35 × 25 cm) under standard laboratory conditions (food and water ad libitum, 20 ± 2 °C, 12 h light/dark cycle starting at 8 a.m., relative humidity 50–60%). Behavioral assessments were performed blind to the experiment in a counterbalanced manner during the light cycle. All procedures of the protocol CEEAH 2481/DMAH 8700 followed Spanish legislation and the EU Directive (2010/63/UE). The study complies with the ARRIVE guidelines developed by the NC3Rs and aims to reduce the number of animals used [22].

### 2.2. Early-Life Intervention: Neonatal Handling

**Figure 1:**
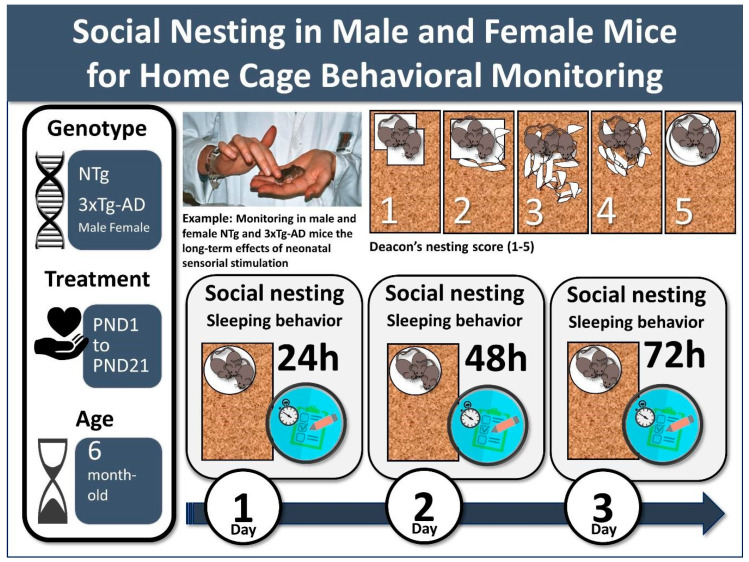
Social nesting in six-month-old male and female mice for home-cage behavioral monitoring in normal (NTg, non-transgenic) and AD-pathological aging (3xTg-AD mice), and the assessment of the long-term effects of neonatal tactile-proprioceptive sensorial stimulation administered from postnatal day 1 to 21.

Early postnatal handling [15] was administered three times every 8 min, twice a day from postnatal day 1 (PND1) to PND21, in half of a set of litters of 6–8 pups of a concurrent breeding program. The procedure consisted of removing the pups from their cotton nest and mother and placing them individually in plastic compartments lined with soft paper towels where they were softly handled and received four tactile stimulations on their back (see Figure 1). This process was repeated. In the control groups, the pups were left undisturbed except for weekly cage cleaning.

### 2.3. Behavioral Assessments

Home-cage sleeping behavior and social nesting construction were evaluated at six months of age. In one of the weekly housing routines, the home-cages with clean sawdust bedding were supplied with two cotton pieces (50 × 50 × 3 mm, Cotofarma, S.L. Badalona, Spain), the same material they had during ontogeny in their nests. Direct observation during the first day of the test verified that all the animals were involved in nest building. On the next day, 48 and 72 h later, the nests were assessed according to Deacon 5-point ordinal scale from 1 to 5 as follows: 1 = not noticeably touched, 2 = partially torn up, 3 = mostly shredded but often no identifiable site, 4 = identifiable but flat, 5 = perfect or nearby, as illustrated in the original publication [23] and represented in icons in Figure 1.

The animals housed in the same cage received the score of the social nest they contributed. Sleeping or not inside the social nest, and the incidence of animals showing sleeping together huddled and/or dog-piled, as a self-organizing behavior [24], was recorded.

### 2.4. Statistics

Data were expressed as a mean with a standard deviation and a 95% confidence interval. The level of significance was set at 0.05. Since data consisted of discrete values, nonparametric statistical tests used were the Mann–Whitney U test for comparison between two groups for each parameter and Kruskal–Wallis test for global comparison of groups for all the parameters. Fisher’s exact test was used to analyze the incidence.

## 3. Results

**Figure 2:**
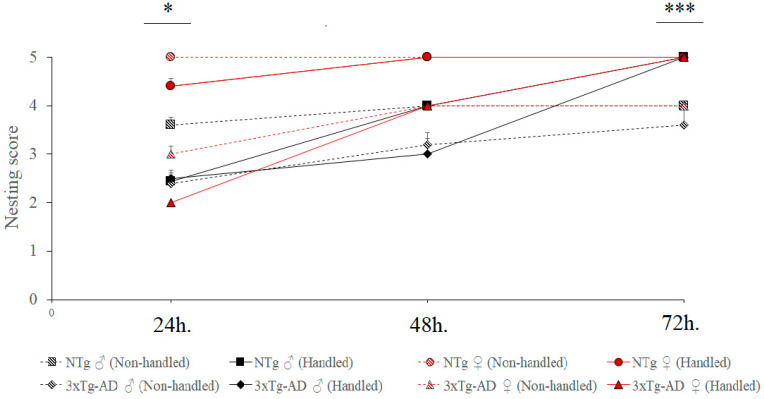
Nesting scores in social structures of male and female six-month-old non-transgenic (NTg) and 3xTg-AD mice using cotton material, and long-term effects of early postnatal handling. Results are expressed as mean ± SEM. *N* = 9–11 per group: Non-handled, male social structure; Handled, male social structure; Non-handled, female social structure; handled, female social structure. Mann–Whitney U test for comparisons, * *p* < 0.05 and *** *p* < 0.001 vs. NTg mice of the same sex.

In all cases, mice slept huddled, curled together inside the social nest, independently of the genotype or sex. As illustrated in Figure 2 and depicted in Table 1, concerning the social nests, in the NTg genotype, females obtained a score of 5 for perfect nests built already at 24 h, whereas male’s nests were mostly shredded but often with no identifiable site (score 3) and the maximum score achieved three days later was 4. In the 3xTg-AD mice, nest building was impaired, with nests at 24 h being partially torn up (score 2) in males and mostly shredded but often with no identifiable site (score 3) in females. The biphasic temporal pattern shown by 3xTg-AD females allowed them to reach a maximum score of 4 at 48 h, but statistically significant genotype difference persisted in all the time intervals (all U = 0.00, *p* < 0.001). Male 3xTg-AD groups progressively reached their maximum score of 3 at 72 h, and statistically significant genotype differences were shown at 24 h (*U* = 8.0, *p* = 0.001) and 48 h (*U* = 20.0, *p* = 0.005). Thus, the male sex’s poor ability to build nests, as previous work already showed in individual subjects, was extensible to social collaboration in nest building.

Early postnatal handling resulted in 100% of NTg and 3xTg-AD mice groups building perfect nests at 72 h, although the temporal performance was sex and genotype-dependent. The progressive improvement to reach perfect nest in handled animals was faster in NTg males. Thus, genotype differences in the male-handed groups could only be observed at 48 h (*U* = 22.5, *p* = 0.016). In handled females, differences were found at 24–48 h (*U* = 0.00, *p* < 0.001) and 72 h (*U* = 35.0, *p* < 0.05). Thus, a maximum score of 5 was achieved in 100% cases at 72 h.

The Kruskal–Wallis test used for global comparison between groups of males (NTg and 3xTg-AD, Non-handled) and (NTg and 3xTg-AD, Handled) for at time nesting construction showed a significant difference of (*p* < 0.01) in the three intervals. See also supplementary File S1 for Wilcoxon–Mann–Whitney U test for early neonatal handling treatment effects. These results are similar to those of female groups, showing a significant difference (*p* < 0.001) in all intervals. Overall, sex- and genotype-dependent temporal ranks were observed as follows: handled NTg females (48 h, score 5) < handled NTg males (48 h, score 4) = handled 3xTg-AD female (48 h, score 4) < handled 3xTg-AD male (48 h, score 3).

## 4. Discussion and Conclusions

The present brief report proposes home-cage social nesting for behavioral and animal welfare monitoring. Also, we provide an example of its use to show long-term effects of neonatal sensorial stimulation in male and female six-month-old adult 3xTgAD mice compared to sex- and age-matched NTg counterparts.

As reported in NTg and 3xTg-AD mice in a setting without nesting material [25], all the animals slept together, huddled inside the nest. While this self-organizing behavior is broken in mice with social interaction and sensorimotor gating abnormalities modeling psychotic-like symptoms [24] or related to sickness behavior [9], the sleeping behavior parameter was not informative in the present study.

Despite three or four animals per cage collaborating to build the social nest, the scores were lower than previously reported in six-month-old single NTg and 3xTg-AD mice and breeding structures using cotton, see [7]. In agreement, our most recent work showed isolated male 3xTgAD mice building better nests than those under standard conditions [26]. Here, scores closely resembled those recorded when using paper towels [7], a nesting material demanding better fine motor functions to be gutted than cotton or other more natural nesting materials that help mice build better nests [27].

Despite the fact that we demonstrated that paper tissue was better than cotton to unveil genotype effects, in the current work, we used cotton. The rationale is because the experimental design involved the early-life experience of being grown in a nest plus an early-life intervention that in its protocol uses soft-paper tissue as an artificial nest [15,18]. Therefore, to unify the “nesting” early-life experiences and to prevent mixing them with the behavioral assessment in adulthood, the best and easiest nesting material [27] was provided to the mothers and used again to assess the animals in their adulthood.

Interestingly, NTg females exhibited perfect performance from the first day, and all the groups of handled animals did so at 72 h. In contrast, males’ poor ability to build individual nests [7] was also shown here. We hypothesize that social collaboration to build a nest among males, mostly in the socially impaired 3xTg-AD mice [25,28], could require more time. Lower protective or thermoregulatory needs due to males’ higher body weight and/or group-sleeping behavior could be another explanation, in agreement with our recent study on isolation [26]. Nesting behavior in the Tg2576 mice is also disrupted [29,30], but in the APPswe/PS1 mice, the impairment is only observed in group-housed conditions [31].

The present work is the first to provide evidence of the long-lasting effects of neonatal handling in a social context. We provide proof-of-concept of social nesting sensitivity to this intervention administered during ontogeny. Despite the current report being limited to two readouts (sleeping and nesting behaviors), in a previous report [15], we demonstrated that this set of 80 animals’ AD-behavioral phenotype was modified. Bizarre or disruptive behaviors, characterized for the first time as early neuropsychiatric-like symptoms in male and female 3xTg-AD mice at six months of age, differed from those exhibited by their age- and sex-matched NTg counterparts. Increased freezing, delayed thigmotaxis, and enhancement of emotional behaviors were also early neuropsychiatric-like symptoms. Reduction of freezing and most of the bizarre or disruptive behaviors, potentiation of risk assessment and horizontal locomotor activity, but not the modification of vertical exploratory activity, demonstrated bidirectional and selective behavioral long-lasting effects of postnatal handling.

The 100% positive results obtained in social nesting at 72 h in handled animals are remarkable. More importantly, these effects of tactile and proprioceptive sensorial stimulation on social behavior were observable six months later, in adulthood. Additionally, the sex- and genotype-dependent temporal ranks to achieve the maximum score, with females and NTg mice building better communal nests, are also interesting to note and suggest distinct biological-psychological-social factors interplay underlying nest building that can be modified by several factors, from intrinsic sex and genetics to cycle of life experiences and environmental factors [32,33,34].

Although in the current experimental design the brains were not studied, we have recently shown in old male NTg and 3xTg-AD mice that the nesting building endpoint at 72 h correlates with the loss of hippocampal size associated with AD-genotype and its worsening by social-isolation [26], although no correlations were found with the levels of hippocampal tau pathology. Further studies are needed to establish other meaningful correlates between nesting behavior and brain areas related to this instrumental task involving fine motor and processes skills.

The measurement reliability of Deacon’s scale is well-recognized [22]. Still, home-cage-based social nesting analysis can hardly be constrained by the group’s statistical power limitation vs. individual recordings. Another aspect to discuss is the score of social nesting being attributed to all the members involved (as verified by direct observation) in the task [14], similarly to what is done when academic marks are attributed to students involved in a group assignment. Except for female NTg mice, none of the groups built a perfect nest during the first day. Therefore, the poorest involvement of one mouse would result in a lower social nesting score. Experimental designs will always be subordinated to behavioral individual screening and the rules to reduce the number of animals used [22].

In our previous scientific report in 12-month-old 3xTg-AD and NTg female mice, social nesting complemented the primary individual behavioral screening of dietary supplementation benefits [14]. In that work, the quality of NTg females’ nests scored 4, while 3xTg-AD females scored 2, lower scores than those reported here at six months of age. Additionally, chronic treatment with selenium did not modify the quality of NTg females’ nests but successfully rescued those built by 3xTg-AD female, achieving a score of 4 at 24 h. In the current experimental design, with a sample size of 80 animals distributed in 8 experimental groups, social nesting results were in agreement with the cognitive and neuropsychiatric-like profiles shown by these animals [15]. The 3-days nesting protocol, assessing the same cage during three consecutive days [7], rather than the classical 24 h scoring [14], can be troubleshooting, since it provides more experimental units to confirm conclusions in social nesting.

In summary, despite the limitations of sample size, social nesting was sensitive to genotype (worse in 3xTg-AD mice), sex (worse in males), profile, and treatment (distinct temporal patterns, time to observe the maximum score and incidence of the perfect nest). The results suggest that social nesting can be easily included in housing routines, monitoring disease progress, and adding a social dimension value in evaluating the potential risk factors and effects of preventive/therapeutical strategies. Social nesting was also sensitive to detect daily life activity patterns in standard wild-type mice and support the benefit of preventive (present work) and therapeutical [14] treatments successfully improving the cognitive, neuropsychiatric- and BPSD (Behavioral and Psychological Symptoms associated to Ddementia)-like phenotype of 3xTg-AD mice. Finally, the noninvasive, painless, simple, short, and low-cost features of this home-cage monitoring are advantages that make social nesting feasible to be implemented in most animal department settings.

## Figures and Tables

**Table 1:** Long-term effects of early postnatal handling on home-cage-based social nests built by male and female six-month-old NTg and 3xTg-AD mice.

Nesting Score in Social Structures, Using Cotton	Time (h) to Observe the Maximum Score	Perfect Nest (Score 5) at 72 h	Incidences % of Score 5 at 72 h
**Non-handled** NTg, male (*n* = 10 mice/3 cages)	72 h	No	0
NTg, female (*n* = 10 mice/3 cages)	24 h	Yes	100
3xTg-AD, male (*n* = 10 mice/3 cages)	72 h	No	40 ***
3xTg-AD, female (*n* = 10 mice/3 cages)	72 h	No	0 ***
**Handled** NTg, male (*n* = 9 mice/3 cages)	72 h	Yes	100^nH^
NTg, female (*n* = 10 mice/3 cages)	48 h	Yes	100
3xTg-AD, male (*n* = 10 mice/3 cages)	72 h	Yes	100^nH^
3xTg-AD, female (*n =* 10 mice/3 cages)	72 h	Yes	100^nH^

Fisher’s exact test, the incidence of Deacon’s nesting score 5, *** *p* < 0.001 vs. NTg genotype. ^nH^ *p* < 0.001 handled vs. non-handled (same genotype).

## Data Availability

The data presented in this study are available on request from the corresponding author.

## Supplementary Materials

The following are available online at https://www.mdpi.com/article/10.3390/ani11041079/s1, File S1: Long-term effects of early-neonatal tactile-proprioceptive handling treatment (PND1-PND21) measured in males and females with normal and AD-pathological aging at 6 months of age.
