# Supplementary material for: Social Nesting, Animal Welfare, and Disease Monitoring
Source: Animals (Basel). 2021 Apr 9;11(4):1079. doi: 10.3390/ani11041079 (PMC8070261; doi:10.3390/ani11041079)
Supplement: Supplementary file 1 [file animals-11-01079-s001.pdf]

Long-term effects of early-neonatal tactile-proprioceptive handling treatment
(PND1-PND21) measured in males and females with normal and AD-pathological aging at 6 months of age

The Wilcoxon-Mann-Whitney U test

Nesting at 24h

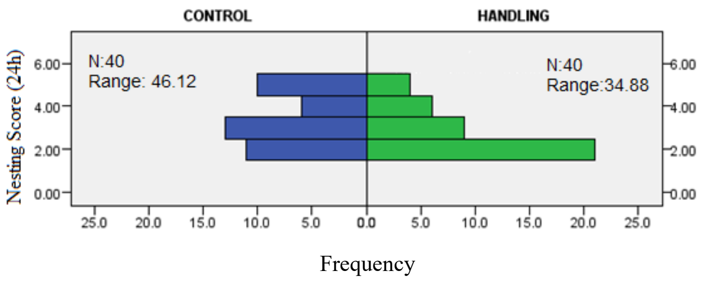

N	80
The Mann-Whitney U test	575.000
The Wilcoxon test	1,395.000
The standard error (SE)	98.951
Asymptotic Sig (bilateral)	0.023

Nesting at 48h

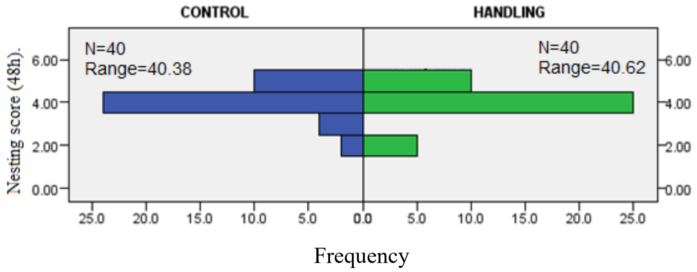

N	80
The Mann-Whitney U test	805.000
The Wilcoxon test	1,625.000
The standard error (SE)	90.235
Asymptotic Sig (bilateral)	0.956

Nesting at 72h

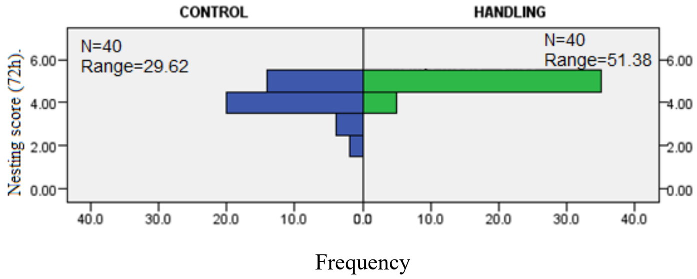

N	80
The Mann-Whitney U test	1,235.000
The Wilcoxon test	2,055.000
The standard error (SE)	89.378
Asymptotic Sig (bilateral)	0.000
